# Cancer gene profiling in non-small cell lung cancers reveals activating mutations in JAK2 and JAK3 with therapeutic implications

**DOI:** 10.1186/s13073-017-0478-1

**Published:** 2017-10-30

**Authors:** Shuyu D. Li, Meng Ma, Hui Li, Aneta Waluszko, Tatyana Sidorenko, Eric E. Schadt, David Y. Zhang, Rong Chen, Fei Ye

**Affiliations:** 1 Department of Genetics and Genomic Sciences, Icahn Institute for Genomics and Multiscale Biology, Icahn School of Medicine at Mount Sinai, New York, NY 10029 USA; 2 Sema4, a Mount Sinai venture, Stamford, CT 06902 USA; 3 Department of Pathology and Laboratory Medicine, Icahn School of Medicine at Mount Sinai, New York, NY 10029 USA; 4 Department of Pathology, New York Medical College, Valhalla, NY 10595 USA

**Keywords:** Non-small cell lung cancer, Cancer hotspot panel, Clinical sequencing, JAK2, JAK3, Immunotherapy

## Abstract

**Background:**

Next-generation sequencing (NGS) of cancer gene panels are widely applied to enable personalized cancer therapy and to identify novel oncogenic mutations.

**Methods:**

We performed targeted NGS on 932 clinical cases of non-small-cell lung cancers (NSCLCs) using the Ion AmpliSeq™ Cancer Hotspot panel v2 assay.

**Results:**

Actionable mutations were identified in 65% of the cases with available targeted therapeutic options, including 26% of the patients with mutations in National Comprehensive Cancer Network (NCCN) guideline genes. Most notably, we discovered *JAK2* p.V617F somatic mutation, a hallmark of myeloproliferative neoplasms, in 1% (9/932) of the NSCLCs. Analysis of cancer cell line pharmacogenomic data showed that a high level of *JAK2* expression in a panel of NSCLC cell lines is correlated with increased sensitivity to a selective JAK2 inhibitor. Further analysis of TCGA genomic data revealed *JAK2* gain or loss due to genetic alterations in NSCLC clinical samples are associated with significantly elevated or reduced PD-L1 expression, suggesting that the activating *JAK2* p.V617F mutation could confer sensitivity to both JAK inhibitors and anti-PD1 immunotherapy. We also detected *JAK3* germline activating mutations in 6.7% (62/932) of the patients who may benefit from anti-PD1 treatment, in light of recent findings that *JAK3* mutations upregulate PD-L1 expression.

**Conclusion:**

Taken together, this study demonstrated the clinical utility of targeted NGS with a focused hotspot cancer gene panel in NSCLCs and identified activating mutations in *JAK2* and *JAK3* with clinical implications inferred through integrative analysis of cancer genetic, genomic, and pharmacogenomic data. The potential of *JAK2* and *JAK3* mutations as response markers for the targeted therapy against JAK kinases or anti-PD1 immunotherapy warrants further investigation.

**Electronic supplementary material:**

The online version of this article (doi:10.1186/s13073-017-0478-1) contains supplementary material, which is available to authorized users.

## Background

Lung cancer remains the most prevalent cancer and the leading cause of cancer mortality with an estimated 222,500 new cases and 156,000 deaths in the United States in 2017 [1]. There are two major histological classes of lung cancers: non-small-cell lung cancer (NSCLC) accounting for approximately 85–90%; and small-cell lung cancers (SCLC) accounting for 10–15%. NSCLC is further classified into several subtypes, with adenocarcinoma and squamous cell carcinoma being the two main subclasses [2]. The genetic landscape of lung cancers has been extensively characterized by cancer genomic sequencing studies including those from the Cancer Genome Atlas (TCGA) research network [3–6]. For example, comprehensive molecular profiling of 230 lung adenocarcinomas confirmed *TP53*, *KRAS*, and *EGFR* as the most frequently mutated genes and identified 15 other significantly mutated genes, including oncogenes *BRAF*, *MET*, and *PIK3CA* and tumor suppressors *STK11*, *KEAP1*, *NF1*, *RB1*, and *CDKN2A* [4]. At the molecular pathway level, RTK/RAS/RAF, PI3K-mTOR, and cell cycle pathways are the most frequently altered in lung adenocarcinomas [4]. In addition, the differences of genomic alterations in smokers and non-smokers have also been investigated [5, 7].

Genetic and genomic profiling in lung cancers have not only facilitated our understanding on the underlying molecular mechanisms of disease pathogenesis, but also significantly impacted clinical practice. The treatment paradigm for NSCLCs has been evolving rapidly due to new therapeutic options and implementation of genetic testing in clinic. National Comprehensive Cancer Network (NCCN) clinical practice guidelines (https://www.nccn.org/professionals/physician_gls/f_guidelines.asp) recommend genetic testing for seven genes (*EGFR*, *ALK*, *ROS1*, *RET*, *BRAF*, *MET*, *HER2*) with available targeted therapies. The matched targeted therapy is the recommended first-line option for non-small cell, non-squamous lung cancer patients who are positive for sensitizing *EGFR* mutations, *ALK* rearrangement, or *ROS1* rearrangement. Emerging evidence has also demonstrated clinical benefit to therapies against BRAF [8–11], MET [12–14], RET [15, 16], or HER2 [17, 18] in patients harboring activating mutations in the corresponding targets. The clinical utility of cancer genomic profiling in NSCLCs has been demonstrated by a recent report of 6800 cases utilizing the FoundationOne® panel (http://foundationone.com/) to facilitate implementation of the NCCN guidelines for lung cancer biomarker testing [19]. The study identified 39% of the tested patients harbor mutations in at least one of the seven genes shown in NCCN guideline [19].

In this study, a total of 932 NSCLC formalin fixed paraffin embedded (FFPE) samples were analyzed to detect various mutations in 50 cancer-related genes using the Ion AmpliSeq™ Cancer Hotspot panel v2 (CHPv2) by targeted next-generation sequencing (NGS). In addition to reporting mutations in the NCCN guideline genes for therapeutic recommendations, our study had three additional major objectives. First, we identified actionable mutations in non-NCCN guideline cancer genes that may guide the patients to enroll clinical trials of the matched targeted therapies, for example the NCI MATCH basket trial [20]. Second, we wanted to explore if there are previously well-characterized oncogenic mutations in other solid tumors or hematological malignancies but have not been described in NSCLCs. Although these mutations are likely extremely rare in NSCLCs, those patients harboring the mutation may benefit from off-label use of available targeted therapies approved in other tumor types. Finally, we also analyzed germline mutations with potential clinical implications.

## Methods

### Tissue samples

The study was approved by the Mount Sinai Institutional Review Board (IRB). Tissue samples, collected during surgical resection or biopsy from May 2015 to March 2017, are sequenced by the molecular pathology lab at Mount Sinai Hospital as part of routine diagnostic workup. A total of 932 NSCLC FFPE samples were included in this retrospective analysis. The majority (>98%) of patients were previously untreated when the tumor samples were collected.

### DNA extraction and quantification

Samples were fixed in formalin, embedded in paraffin, then sectioned at 5-μm thickness. Regions of tissue containing tumor cells, identified by hematoxylin and eosin-stained sister slides, were scraped and processed for DNA extraction. FFPE specimens were used. The Maxwell 16 FFPE Tissue LEV DNA Kit (Promega) was used for DNA extraction from the tissue sections according to the manufacturer’s instructions. The concentrations of extracted DNAs were measured and quantified using the Qubit Fluorometer system (Life Technologies). The average DNA concentration is 27.3 ng/μL (range 0.25–374).

### AmpliSeq hotspot cancer panel v2 by NGS

NGS: typically, 30 ng (range 3.6–35 ng) of DNA from each sample was used to prepare barcoded libraries using Life Technology’s Ion AmpliSeq™ Cancer Hotspot Panel v2, IonXpress barcoded adapters, and Ion AmpliSeq Library Kit 2.0 (Life Technologies). This panel consists of 207 amplicons covering over 20,000 bases of 50 genes with known cancer associations. The genes included in this panel are *ABL1*, *AKT1*, *ALK*, *APC*, *ATM*, *BRAF*, *CDH1*, *CDKN2A*, *CSF1R*, *CTNNB1*, *EGFR*, *ERBB2*, *ERBB4*, *EZH2*, *FBXW7*, *FGFR1*, *FGFR2*, *FGFR3*, *FLT3*, *GNA11*, *GNAS*, *GNAQ*, *HNF1A*, *HRAS*, *IDH1*, *IDH2*, *JAK2*, *JAK3*, *KDR*, *KIT*, *KRAS*, *MET*, *MLH1*, *MPL*, *NOTCH1*, *NPM1*, *NRAS*, *PDGFRA*, *PIK3CA*, *PTEN*, *PTPN11*, *RB1*, *RET*, *SMAD4*, *SMARCB1*, *SMO*, *SRC*, *STK11*, *TP53*, *VHL*. Details on the molecular functions and their relevance to cancers are provided in Additional file 1: Table S1. Each library was barcoded with the Ion Xpress Barcode Adapters 1–16 Kit (Life Technologies). The Ion Library Equalizer Kit was used to normalize library concentration to 100 pM. Barcoded Ampliseq libraries were pooled, combined to a final concentration of 20 pM, subjected to emulsion polymerase chain reaction (PCR) and enrichment using the OneTouch2 and OneTouch ES instrument and then sequenced on the Ion Torrent PGM (12 samples per 318 IonChip). Data analysis was carried out with Torrent Suite Software V.4.0.2 (Life Technologies) and alignment to the hg19 human reference genome.

### Quantification of JAK2 p.V617F mutation by real-time quantitative polymerase chain reaction (qRT-PCR)

*JAK2* p.V617F mutation allele burden was carried out by allele specific RT-PCR on LightCycler 480 instrument (Roche). Wild type (WT) and mutated alleles were detected in two separate reactions for the same sample. Briefly, two RT-PCRs were performed in parallel with a common forward primer (5′-TTATGGACAACAGTCAAACAACAAT-3′) and only differed in the use of a reverse primer specific for the *JAK2* WT and the p.V617F mutated DNA, respectively (Reverse-WT: 5′-TTTACTTACTCTCGTCTCCACAGtC-3′; Reverse-V617F: 5′-TTTACTTACTCTCGTCTCCACAGtA-3′). Reactions were performed using the following PCR conditions: 95 °C for 10 min for initial denaturation, followed by ten cycles of 15 s at 95 °C, 1 min at 65 °C (decreasing 0.7 °C per cycle), and followed by 45 cycles of 15 s at 95 °C, and 1 min at 58 °C. The *JAK2* p.V617F proportion was calculated from cycle threshold (CT). Every sample has a value of ΔCT, which is the difference of the values of CT between the two primers. The ratio of *JAK2* p.V617F mutation to WT was 2^–ΔCT^. The proportion of *JAK2* p.V617F mutation was that 2^–ΔCT^ was divided by 1 + 2^–ΔCT^. The sensitivity of detecting the *JAK2* p.V617F mutation by this assay was 0.05%.

### Analysis of publicly available cancer genomic and pharmacogenomic data

The Genomics of Drug Sensitivity in Cancer (GDSC) data were downloaded from http://www.cancerrxgene.org/downloads. For correlation analysis between gene expression and drug sensitivity, RMA normalized gene expression values based on Affymetrix Human Genome U219 arrays and log-transformed half maximal inhibitory concentration (IC50) values for all screened cell line/drug combinations were used. To compare gene expression between sensitive and resistant cell lines, a two-tailed *t*-test was used.

The TCGA sequencing data for NSCLCs were downloaded from the TCGA data portal (https://tcga-data.nci.nih.gov/docs/publications/tcga/?). Log-transformed relative standard error of the mean (RSEM) values from RNA-sequencing (RNA-seq) data were used for gene expression analysis. Somatic mutation and copy number variation results for genes of interest were downloaded from cBioPortal (http://www.cbioportal.org/) [21, 22]. To compare gene expression between patients harboring various genetic alterations, pairwise two-tailed *t*-tests were performed.

## Results

### Clinicopathological characteristics of patient samples

From May 2015 to March 2017, 985 patients were enrolled and 932 were eligible. The majority (>98%) of patients were previously untreated when the tumor samples were collected. The major reasons for ineligibility of the 53 cases were either insufficient tumor cells (<10%) or lack of DNA amount for NGS panel testing. Among the 932 patients with NGS testing, the median age was 67 years (age range 36–90 years) and 57% of the patients were women. Most patients were former smokers and 24% were never smokers. With respect to histological subtypes, the majority of the cases in the cohort (98%) were NSCLCs and 85% had stage III/IV disease at diagnosis. The mean sequencing depth for the 932 samples across the 207 amplicons was 2129x, with overall very high percentage of reads on target (>95%) and very good uniformity (>90% above 0.2x mean base read depth). The 200x nucleic acid coverage and 2% of mutation allele fraction were used as the cutoff to make the final variant call. All mutations with allele fractions < 5% were confirmed by a secondary assay.

### Actionable mutations with available targeted therapies

We identified a total of 2898 mutations in the 932 tumor samples. Details of these mutations including chromosome locations, WT and mutant alleles, mutation allele fractions, coding nucleotide sequence, and amino acid changes are provided in Additional file 1: Table S2. We first examined activating mutations in the seven NCCN guideline genes (Table 1). *EGFR* mutations are detected in 231 of the 932 patients. Of these patients, 188 (20%, 188/932) harbor sensitizing mutations and are recommended for first-line treatment with gefitinib, erlotinib, or afatinib by the NCCN guideline. There are 14 (1.5%, 14/932) patients who harbor the acquired resistance mutation p.T790M and would be amenable to third-generation EGFR inhibitors such as osimertinib [23] for second-line or subsequent treatment. There are exon 20 insertions in 22 patients who are resistant to EGFR tyrosine kinase inhibitors (TKIs) and currently there is no effective targeted treatment available. The rest of the seven mutations in *EGFR* have unknown functional significance. *BRAF* is mutated in 34 patients: 19 (2.0%, 19/932) are activating mutations with vemurafenib [8, 9] or dabrafenib (with or without MEK inhibitor trametinib) [10, 11] as treatment options; nine (1.0%, 9/932) mutations impaired BRAF functions and the patients may respond to dasatinib [24]; the remaining six mutations have unknown functional consequences. Exon 20 insertion in *HER2* (*ERBB2*) is identified in 16 (1.7%, 16/932) patients and available targeted agents include trastuzumab or afatinib [17, 18]. Due to the limitation of the CHPv2 panel, we were unable to assess *ALK* fusion, *ROS1* fusion, *RET* fusion, and *MET* exon 14 skipping mutations in our study. No activating missense mutations were discovered in these four genes by the panel. In total, 246 (26%, 246/932) patients in this cohort harbor an actionable mutation in the NCCN guideline genes with treatment recommended by the guideline.

**Table 1:** Mutated cancer genes and available targeted therapeutics

Gene	Mutation frequency in this study, n (%)	Actionable mutations, n (%)	Targeted therapy
EGFR^a^	231 (25)	202 (22)	EGFR TKIs
BRAF^a^	34 (3.6)	28 (3.0)	Vemurafenib, dabrafenib
HER2^a^	16 (1.7)	16 (1.7)	Afatinib, trastuzumab
KRAS, HRAS, NRAS	298 (32)	298 (32)	MEK inhibitors
CDKN2A	38 (4.1)	38 (4.1)	Cell cycle inhibitors
IDH1, IDH2	6 (0.64)	6 (0.64)	IDH inhibitors
PIK3CA, PTEN	55 (5.8)	55 (5.8)	PI3 kinase inhibitors
ATM	39 (4.2)	39 (4.2)	PARP inhibitors
STK11	26 (2.8)	26 (2.8)	mTOR inhibitors

^a^NCCN guideline genes

Next, we investigated non-NCCN guideline genes for actionable mutations with available targeted therapies currently in clinical trials for NSCLC indications (Table 1). *KRAS*, *HRAS*, and *NRAS* are mutated in 292, two, and five cases, respectively, with a total of 298 cases (32%, 298/932; one patient carries both *KRAS* p.G12S and *NRAS* p.G12A mutation), and these patients may choose to enroll MEK inhibitor trials. *CDKN2A* is mutated in 38 patients (4.1%, 38/932) and multiple trials for cell cycle inhibitors are recruiting, for example a phase II study (NCT02478320, https://clinicaltrials.gov) of aurora kinase inhibitor ilorasertib in CDKN2A deficient solid tumors. Activating mutations in *IDH1* and *IDH2* are detected in six patients (0.64%, 6/932) who are eligible for clinical trials such as NCT02746081 (https://clinicaltrials.gov). *PIK3CA* and *PTEN* are mutated in 42 (4.5%, 42/932) and 13 (1.4%, 13/932) patients, respectively, and NCI MATCH study has sub-protocols (EAY131-I, EAY131-N) for these patients. Mutations including both germline and likely somatic are also identified in *ATM* (39 cases; 4.2%, 39/932) and *STK11* (26 cases; 2.8%, 26/932). The patients may enroll in clinical studies of PARP inhibitors (for *ATM* mutation) or mTOR inhibitors (for *STK11* mutations). In total, 65% of the patients in our study harbor actionable mutations in either NCCN guideline genes or one of above described genes with available targeted therapeutic options (Table 1). Unfortunately, we do not have follow-up information from the treating oncologists on whether the patient indeed received the matched targeted therapy or enrolled in clinical trials. We speculate that the patients are more likely to have received the therapy if sensitizing mutations in the NCCN guideline genes (e.g. EGFR and BRAF), with the targeted therapy approved by the Food and Drug Administration (FDA) for NSCLC, were detected and the patient’s performance status allows. For other genes, the matched therapy is based on molecular mechanisms and limited clinical evidences, and the attending physician may or may not have prescribed the targeted drugs for off-label use or recommended the patient to enroll in clinical trials if available.

### JAK2 p.V617F oncogenic mutation in 1% of NSCLCs

While the primary objective of clinical sequencing of cancer gene panels is to identify targetable mutations that have been previously characterized in a specific tumor type in order to guide therapeutic strategies, accumulation of large volume sequencing data from a specific tumor type would also enable discovery of novel oncogenic mutations that have not been well-described in the tested tumor type. We interrogated the sequencing data in the current study and identified *JAK2* p.V617F mutation in nine patients (1.0%, 9/932; Table 2). *JAK2* p.V617F is an activating mutation frequently detected in myeloproliferative disorders (MPN), specifically in > 90% of patients with polycythemia vera and in 60% of patients with essential thrombocythemia or idiopathic myelofibrosis [25–27]. The *JAK2* p.V617F mutation causes constitutive activation of JAK2 kinase and consequently JAK-STAT signaling pathway. The JAK-STAT pathway regulates cellular processes including proliferation, differentiation, and apoptosis, and its role in tumorigenesis and cancer development has been well documented for both hematological malignancies [28, 29] as well as solid tumors [30]. Inhibitors of JAK kinases have been developed to treat those cancers that aberrant JAK-STAT pathway activity is a major mechanism of disease pathogenesis [31]. Ruxolitinib, a small molecule inhibitor of JAK1 and JAK2 kinases, has been approved by the FDA for the treatment of polycythemia vera and intermediate to high-risk myelofibrosis.

**Table 2:** *JAK2* p.V617F mutation in nine NSCLCs. Co-occurrence with well-characterized NSCLC oncogenic mutations in *KRAS*, *EGFR*, and *BRAF* are shown

Sample ID	Tumor type	Tumor cell (%)	Sequencing depth (x)	JAK2 p.V617F allele fraction (%)	Co-occurring mutation (allele fraction %)
592	Non-small cell carcinoma	30	1999	8.2	KRAS p.G12V (17.2)
664	Adenocarcinoma with squamous features	50	1996	7	BRAF p.V600E (27.3)
717	Adenocarcinoma	40	1273	2	-
915	Favoring adenocarcinoma	60	1539	2.1	EGFR exon19 del (52.1)
1182	Adenocarcinoma	40	1997	2.4	BRAF p.V600E (10.4)
1200	Mucinous adenocarcinoma	10	1422	10.5	KRAS p.G12V (4.8)
1527	Adenocarcinoma	30	1997	10.8	-
1588	Adenocarcinoma, acinar type	20	892	6.5	-
1825	Adenocarcinoma with mucinous features	30	1998	13.4	KRAS p.G12C (25.4)

**Fig. 1:**
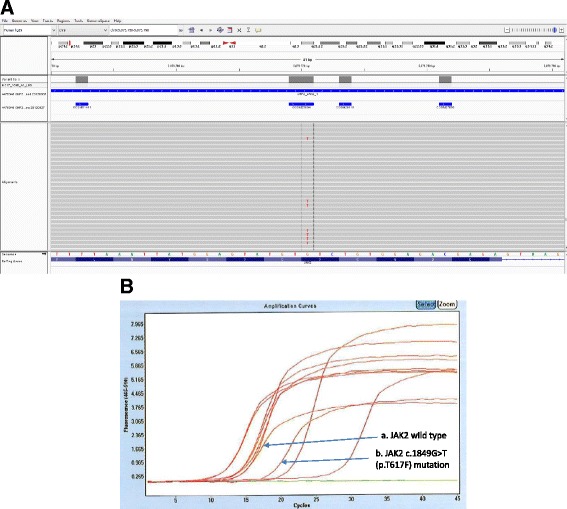
Examples for the detection of *JAK2* p.V617F mutation by targeted NGS and real-time allele-specific PCR. **a** Targeted NGS: IGV view for *JAK2* c.1849G > T (p.V617F) mutation in sample 1825. The sequencing depth at the mutation site is 1998x, with 268 sequencing reads representing the mutant allele (T) and 1730 reads representing the WT allele (G). **b** Real-time allele-specific PCR on LightCycler 480 instrument (Roche). An example for one sample shown in the amplification plot: **a**. results for *JAK2* WT; **b**. results for *JAK2* c.1849G > T (p.V617F). The rest of the curves represent unrelated samples in the PCR reaction

A representative sequence alignment is shown in Fig. 1a illustrating the c.1849G > T mutation in *JAK2* coding region leading to the p.V617F amino acid change. Low mutation allele fraction in the range of 2–13% in the nine tumor samples (Table 2) strongly suggests the mutation is somatic. We subsequently performed real-time allele-specific PCR for *JAK2* p.V617F and confirmed this mutation in all of the nine tumor samples (an example shown in Fig. 1b). We then tested co-occurrence or mutual exclusivity of the *JAK2* p.V617F mutation with other well-characterized lung cancer genes including RAS family genes (*KRAS*, *NRAS*, *HRAS*), *EGFR*, *BRAF*, and *HER2*. As expected, mutations in the *RAS* genes, *EGFR*, *BRAF*, and *HER2*, are largely mutually exclusive. Of the nine cases with the *JAK2* p.V617F mutation, three co-occurred with a *KRAS* mutation, one with a *EGFR* mutation, and two with the *BRAF* p.V600E mutation (Table 2). Further examination of mutation allele fractions indicates the *JAK2* p.V617F mutation has very different allele fractions than the co-occurring oncogenic mutations (Table 2), suggesting they are derived from different subclonal cell populations in the same tumor specimen.

**Fig. 2:**
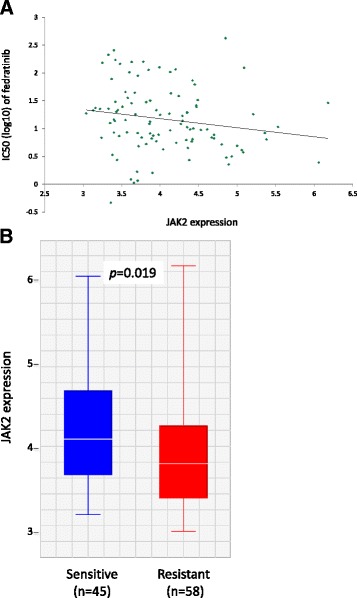
Correlation between *JAK2* expression and sensitivity to a selective JAK2 inhibitor fedratinib in NSCLC cell lines. Gene expression data are RMA normalized (log2-based) values derived from microarray gene expression profiling (see “Methods”). **a** IC50 of fedratinib and *JAK2* expression level in 103 NSCLC cell lines. **b** *JAK2* expression in fedratinib sensitive vs. resistant cell lines

To evaluate the sensitivity of lung cancer cells to pharmacological inhibition of JAK2, we analyzed the GDSC dataset including exome sequencing data, gene expression profiling data, and screening results of 265 cancer drugs in more than 900 cancer cell lines [32, 33], among them 103 are NSCLC cell lines. The NSCLC cell lines are overall resistant to ruxolitinib, with only one cell line having an IC50 < 10 μm. However, we observed greater sensitivity to a selective JAK2 inhibitor fedratinib [34] with almost half of the NSCLC cell lines having an IC50 < 10 μm. Due to the lack of *JAK2* mutations in the 103 NSCLC cell lines, we used *JAK2* expression as an approximation for JAK2 activity to test if it has any association with sensitivity to fedratinib. The result shows a modest correlation between high level of *JAK2* expression and increased sensitivity to fedratinib (Pearson’s correlation coefficient = −0.18; Fig. 2a). When we arbitrarily divided the 103 cell lines into a sensitive group (IC50 < 10 μm) and a resistant group (IC50 > 10 μm), *JAK2* expression is significantly higher in the sensitive group (*p* = 0.019, two-sided *t*-test; Fig. 2b).

**Fig. 3:**
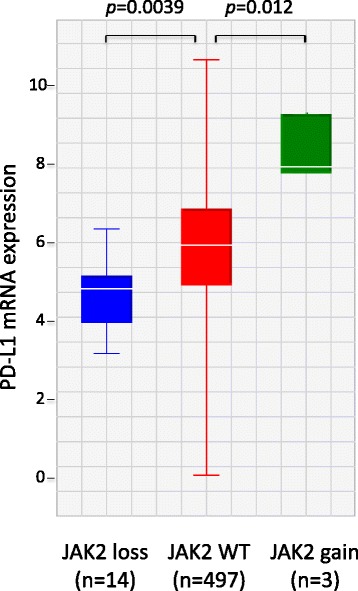
PD-L1 mRNA expression in tumors with *JAK2* gain (amplification) or loss (nonsense mutations or homozygous deletion) in the TCGA cohort. Gene expression data are log2-transformed RSEM values derived from RNA-seq (see “Methods”)

Loss-of-function mutations in *JAK1* and *JAK2* have been associated with primary and acquired resistance to PD-1 blockade in melanomas [35, 36]. To explore the implication of *JAK2* mutations in lung cancers in the context of immunotherapy, we analyzed the TCGA cohort of 515 lung adenocarcinomas with both exome sequencing and RNA-seq data available [5]. While no activating *JAK2* mutation is present in the TCGA cohort, *JAK2* is amplified in three tumors. In addition, inactivating somatic alternations including nonsense mutations and homozygous deletions are identified in 14 out of the 515 tumors (three with nonsense mutations and 11 with homozygous deletions). Gene expression analysis revealed that *JAK2* gain is associated with significantly elevated PD-L1 expression (*p* = 0.012, two-sided *t*-test; Fig. 3) while *JAK2* loss is associated with significantly reduced PD-L1 expression (*p* = 0.0039, two-sided *t*-test; Fig. 3). *JAK2* loss due to either nonsense mutation or deletion had similar effect on PD-L1 expression (Additional file 2: Figure S1). We hypothesize that *JAK2* activating mutations such as p.V617F could be associated with increased PD-L1 expression and therefore sensitize the tumor to anti-PD1 immunotherapy. Although we were unable to directly test this idea due to the lack of PD-L1 expression data, the hypothesis has significant potential clinical impact and warrants future investigations.

### Germline mutations with clinical implications

The 2800 mutational hotspots covered by CHPv2 include both somatic and germline mutations. While the germline mutations are often overlooked by previously published clinical sequencing studies [19], we analyzed our cohort for germline mutations with potential clinical implications. For example, *KIT* p.M541L mutation detected in 146 (16%, 146/932) tumors and *KDR* p.Q472H mutation in 5 (0.5%, 5/932) tumors are associated with resistance to EGFR inhibitors in NSCLCs [37]. The *KDR* p.Q472H germline mutation also confers increased sensitivity to anti-angiogenesis treatment in melanomas [38]. Notably, we observed activating *JAK3* germline mutations p.P132T and p.V722I [39] in a combined 62 patients (6.7%, 62/932; 38 and 25 for p.P132T and p.V722I, respectively, with one patient positive for both mutations). Examination of variant allele fraction (VAF) for these *JAK3* mutations showed that most of the VAFs are close to 50% with median VAF = 51.4% (Additional file 1: Table S3; Additional file 2: Figure S2), more likely supporting germline mutations. In contrast, distribution of VAFs for those well-known somatic mutations in *EGFR* showed a wide spectrum and lower average VAF (Additional file 2: Figure S2). The *JAK3* p.V722I germline mutation has been identified in a lung cancer patient with long-term benefit to anti-PD-L1 antibody atezolizumab [40]. Furthermore, it was shown that the mutant JAK3 protein promoted PD-L1 expression in vitro and PD-L1 positivity is substantially enriched in clinical tumor samples with *JAK3* mutations [40]. Therefore, we predict the 6.7% patients in our cohort may respond to anti-PD1 therapy.

**Fig. 4:**
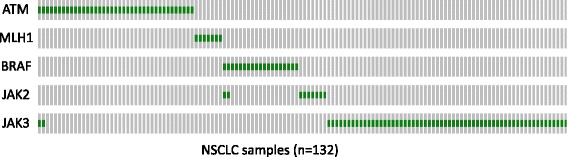
Co-occurrence and mutual exclusivity of *JAK3* germline activating mutation with mutations in *ATM*, *MLH1*, *BRAF*, and *JAK2. Green* and *gray colors* represent mutation and WT, respectively

There are considerable ongoing efforts to identify genetic markers for response to immunotherapy. Several studies have elucidated that DNA mismatch repair deficiency and mutations in genes involved in maintenance of genomic integrity are predictive of durable clinical benefit to immunotherapy [41, 42]. A preliminary report also shows PD-L1 positive lung cancer is enriched for *BRAF* mutations (http://www.abstractsonline.com/pp8/#!/4292/presentation/1306). We tested the relationship between *JAK3* germline mutations and mutations in two DNA repair pathway genes on the panel *ATM* and *MLH1*, *BRAF*, and the *JAK2* p.V617F mutation; our analysis revealed near complete mutual exclusivity among these mutations (Fig. 4), suggesting *JAK3* germline mutation could be a novel, independent genetic marker for responses to anti-PD1 immunotherapy.

## Discussion

The current study demonstrated the clinical utility of NGS-based cancer gene profiling in NSCLCs. A total of 65% patients in our study harbor mutations with available matched targeted therapeutic options, including 26% of patients with actionable mutations in genes recommended for cancer genetic testing by the NCCN guideline. In comparison to a previously published study [19], we not only focused on the clinical application of cancer genetic testing, but also utilized the accumulated data in a large cohort to identify novel oncogenic mutations in NSCLCs. Moreover, we integrated publicly available cancer genetic, genomic, and pharmacogenomic data sources to explore the clinical implication of novel mutations.

One of the most intriguing findings described here is the discovery of the *JAK2* p.V617F mutation in nine NSCLCs, account for approximately 1% of the tested cases. Although it has been reported that this mutation may be detected in around 10% of healthy volunteers, the mutation allele fraction (0.035%) in healthy individuals is far below the clinical threshold (1%) in cancers [43, 44]. We also conducted a thorough clinical review of the nine patients who harbor the *JAK2* p.V617F mutation and none of them had any hematological abnormalities. Therefore, we ruled out the possibilities that the mutation is a technical or clinical artifact. Interestingly, although observed in three cases by an early study [45], the *JAK2* p.V617F mutation was not identified in numerous whole-genome sequencing (WGS) or whole-exome sequencing (WES) studies of thousands of NSCLCs [5, 46–50]. One possible explanation is relatively low sequencing depth by WGS and WES for detection of mutations with low allele fractions. We were able to detect low allele fraction (2–13%) of the *JAK2* p.V617F mutation in our cohort with > 2000x sequencing depth. Another explanation is that majority of the patients in the published genomic sequencing studies are Caucasians or East Asians, while the patients in the current study are from Mount Sinai Hospital and have a high percentage of African Americans and Hispanics.

Although low allele fractions suggest that the *JAK2* p.V617F mutation is from subclonal tumor cells in the tested samples, there is an alternative interpretation pertaining to an aging-related phenomenon referred to as clonal hematopoiesis of indeterminate potential (CHIP) [51]. Defined as the presence of a genetically distinct, hematopoietic stem cell-derived subpopulation of blood cells harboring somatic mutations but without apparent hematological abnormalities, CHIP is common among older individuals and is associated with an increased risk of hematological cancers and cardiovascular diseases [52–54]. It is conceivable the *JAK2* p.V617F mutation we detected is due to infiltrating hematological cells in persons with CHIP. However, we consider this scenario unlikely since the percentage of hematological cells in our sequenced samples is almost zero, in addition to the published results that somatic mutations in leukemia and lymphoma-related genes detected in blood samples of individuals with CHIP have overall low allele fractions [55]. Nevertheless, we recognize there is at least a possibility that for some of the nine patients with the *JAK2* p.V617F mutation in our study, the clonal population in blood due to CHIP may approach 100% and the percentage of hematological cells in the sequenced tumor samples is higher than typical cases, therefore leading to the detection of the *JAK2* p.V617F mutation due to infiltrating hematological cells. Unfortunately, we were not able to sequence the blood samples of the nine patients to rule out this possibility.

The role of the JAK-STAT pathway in lung cancers has been increasingly recognized. Specifically, it has been described that JAK-STAT pathway activity was upregulated in EGFR TKI resistant, EGFR mutant NSCLC cells, and JAK2 inhibition re-sensitizes resistant cells to EGFR TKIs [56, 57]. A recent study also delineated JAK-STAT pathway as a key mediator in lung cancer metastasis [58]. Several clinical trials of JAK inhibitors in NSCLCs are ongoing (NCT02119650, https://clinicaltrials.gov). As *JAK2* p.V617F mutation in NSCLCs was identified in this study, coupled with associations between high level *JAK2* expression and sensitivity to JAK2 inhibition in cell lines, we advocate genetic testing of NSCLCs for the presence of the *JAK2* mutation to determine if it is a response marker to JAK inhibitors in clinic. Furthermore, in light of recent findings on *JAK1* and *JAK2* inactivating mutations as a genetic mechanism for innate as well as acquired resistance to anti-PD1 in melanomas [35, 36], our results suggest *JAK2* activating mutations may serve as a marker for response to both JAK inhibitors and anti-PD1 immunotherapy in NSCLCs.

There is a substantial sub-population in our cohort, 6.7% of the patients with *JAK3* germline activating mutations who may benefit from anti-PD1 treatment. While all of the *JAK3* mutations in the TCGA cohort are p.V722I, 38 of the 62 *JAK3* mutations in our study are p.P132T. This is likely due to high percentage of African Americans in our cohort and the p.P132T (dbSNP accession rs3212723) germline variant is only present in populations of African ancestry with 10% minor allele frequency in the 1000 Genomes Project. Although currently there are not sufficient data to compare responses to immunotherapy among different ethnicities since only 1–2% of the participants in the published immunotherapy clinical trials are African Americans [59, 60], our results suggest African American NSCLC patients may have an overall higher response rate due to ethnicity-specific *JAK3* mutations. However, we should point out that although both p.V722I and p.P132T in JAK3 are activating mutations [39], only p.V722I JAK3 mutant protein has been shown experimentally to promote PD-L1 expression [40]. Although it is implied that the p.P132T mutant protein would also activate PD-L1 expression, it should be directly tested.

Overall, our results demonstrate potential crosstalk between the status of *JAK2/3* mutations and response to JAK2 inhibitors or PD-L1 expression, thus providing a molecular rational for combination of JAK kinase inhibitor therapy and anti-PD1 immunotherapy or combination of JAK inhibition and EGFR targeted TKI therapy in NSCLC patients.

We recognize the limitations of our study. The CHPv2 panel has a limited scope including only mutational hotspots in 50 cancer-related genes. The sequencing assay does not detect gene fusions. Therefore, it requires separate tests to identify several well-known oncogenic events in NSCLCs such as gene fusions involving *ALK*, *ROS1*, and *RET*, and *MET* exon 14 skipping mutations. Although associations between *JAK2* expression and sensitivity to JAK2 inhibition in NSCLC cell lines provided supporting evidence that NSCLCs harboring the activating *JAK2* p.V617F mutation may respond to JAK inhibitors, more thorough preclinical studies, and ultimately clinical studies are required to test the hypothesis. Furthermore, due to the lack of PD-L1 immunohistochemistry (IHC) data in our study cohort, we used TCGA genomic data to correlate *JAK2* genetic alteration with PD-L1 mRNA expression. We note that *JAK2* and the gene encoding PD-L1, *CD274*, are co-localized on chromosome 9p24 therefore confounding this analysis. Although *JAK2* loss due to nonsense mutations or gene deletions had similar effect on PD-L1 expression (Additional file 2: Figure S1), activating mutations in *JAK2* are not present in the TCGA cohort, making it impossible to test if *JAK2* activation due to activating mutations are associated with elevated PD-L1 expression. While our results of correlation between *JAK2* genetic alteration and PD-L1 expression in TCGA genomic data only suggests the *JAK2* p.V617F mutation may correlate with high level PD-L1 expression, a direct analysis of IHC-based PD-L1 expression in tumor samples of NSCLCs carrying the *JAK2* p.V617F mutation is essential when such data become available in the future.

## Conclusion

In conclusion, the current study demonstrated the clinical utility of targeted NGS with a focused hotspot cancer gene panel in NSCLCs, and identified activating somatic mutations in *JAK2* and germline mutations in *JAK3* with clinical implications inferred through integrative analysis of cancer genetic, genomic, and pharmacogenomic data. The potential of *JAK2* and *JAK3* mutations as response markers for the targeted therapy against JAK kinases or anti-PD1 immunotherapy warrants further preclinical and clinical investigations.

## Additional files

Additional file 1: Table S1. List of 50 genes on the Ion AmpliSeq Cancer Hotspot Panel v2. Gene symbol, full name, and a summary from NCBI Entrez Gene database are shown. **Table S2.** The mutations identified in 932 NSCLC tumor samples in this study. **Table S3.** JAK3 germline mutations identified in the study. For mutations in each sample, variant allele fraction (VAF) is shown. (XLSX 197 kb)

Additional file 2: Figure S1. PD-L1 mRNA expression in tumors with *JAK2* loss due to nonsense mutations (JAK2 mut), *JAK2* loss due to homozygous deletion (JAK2 del), *JAK2* wild type (JAK2 WT), or *JAK2* gain due to amplifications in the TCGA cohort. **Figure S2.** Variant allele fraction (VAF) distribution for mutations in *JAK3* and *EGFR*. (PDF 211 kb)

## Abbreviations

CHPv2: Ion AmpliSeq Cancer Hotspot Panel v2
FFPE: Formalin-fixed, paraffin-embedded
GDSC: Genomics of Drug Sensitivity in Cancer
IHC: Immunohistochemistry
MPN: Myeloproliferative neoplasms
NGS: Next-generation sequencing
NSCLC: Non-small-cell lung cancer
SCLC: Small-cell lung cancer
TCGA: The Cancer Genome Atlas
