# Supplementary figures and images for: Cancer gene profiling in non-small cell lung cancers reveals activating mutations in JAK2 and JAK3 with therapeutic implications
Source: Genome Med. 2017 Oct 30;9:89. doi: 10.1186/s13073-017-0478-1 (PMC5662094; doi:10.1186/s13073-017-0478-1)

Figure S1

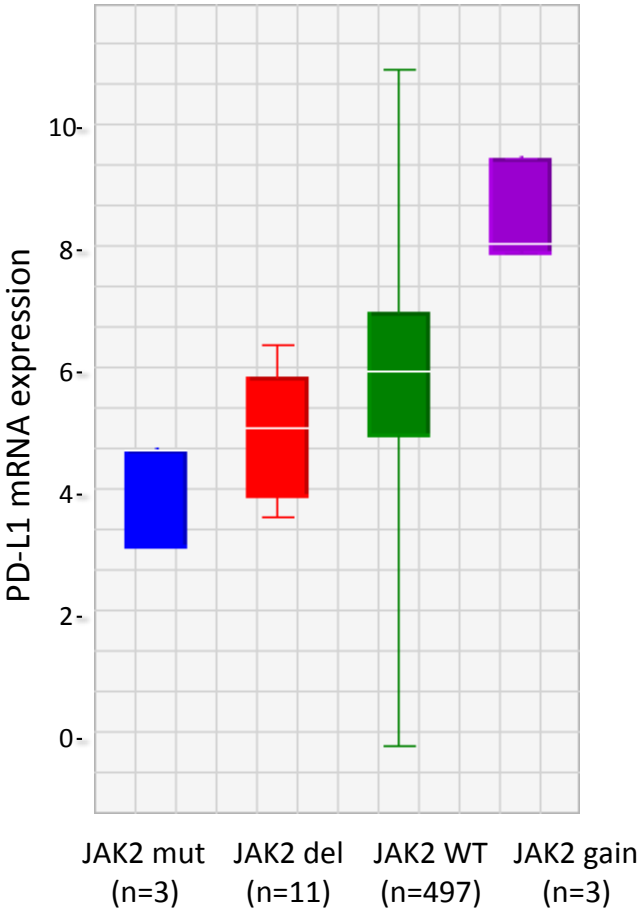

Figure S2

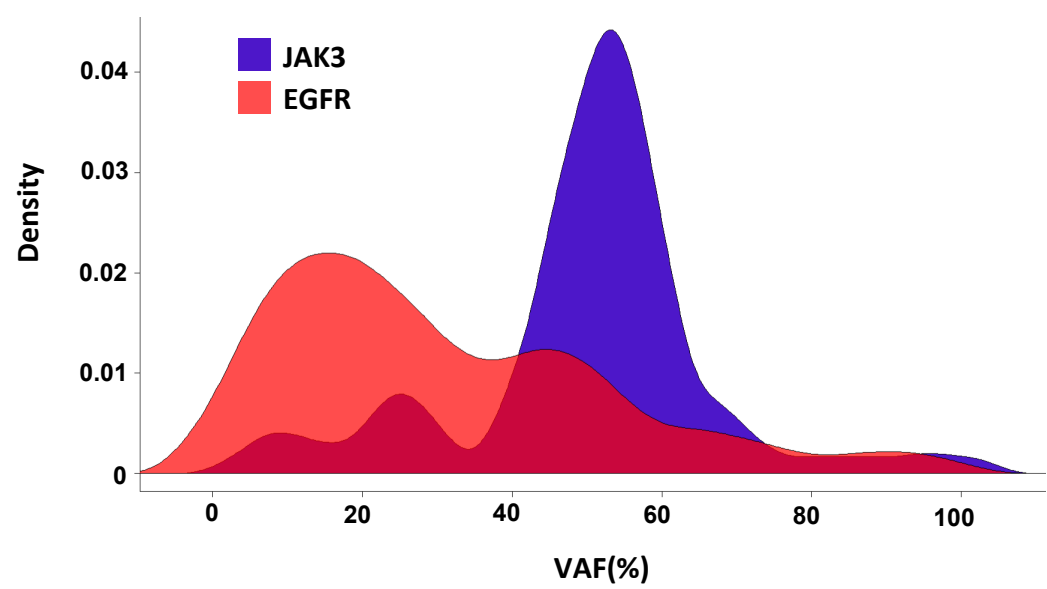

Supplement: Supplementary file 2 — PD-L1 mRNA expression in tumors with JAK2 loss due to nonsense mutations (JAK2 mut), JAK2 loss due to homozygous deletion (JAK2 del), JAK2 wild type (JAK2 WT), or JAK2 gain due to amplifications in the TCGA cohort. Figure S2. Variant allele fraction (VAF) distribution for mutations in JAK3 and EGFR. (PDF 211 kb) [file 13073_2017_478_MOESM2_ESM.pdf]
